# Prediction of an effective cervical ripenning in the induction of labour using vaginal dinoprostone

**DOI:** 10.1038/s41598-023-33974-7

**Published:** 2023-04-26

**Authors:** Nuria López Jiménez, Fiamma García Sánchez, Rafael Hernández Pailos, Valentin Rodrigo Álvaro, Ana Pascual Pedreño, María Moreno Cid, Antonio Hernández Martínez, Milagros Molina Alarcón

**Affiliations:** 1 Department of Obstetrics and Gynecology, Hospital Universitario de Torrevieja, 03186 Torrevieja, Spain; 2 Department of Obstetrics and Gynecology, Hospital General Universitario Nuestra Señora del Prado, 45600 Talavera de la Reina, Toledo, Spain; 3 Department of Nursing, Physiotherapy and Occupational Therapy, Faculty of Nursing, University of Castilla-La Mancha IDINE, 02001 Albacete, Spain; 4 Department of Nursing, Physiotherapy and Occupational Therapy, Faculty of Nursing, University of Castilla La Mancha IDINE, 13071 Ciudad Real, Spain; 5 Department of Obstetrics and Gynecology, Hospital La Mancha Centro, 13600 Alcázar de San Juan, Ciudad Real, Spain; 6 Department of Gynecology, Hospital Universitario Ramón y Cajal, 28034 Madrid, Spain

**Keywords:** Health care, Medical research, Risk factors

## Abstract

To develop a predictive model for successful cervical ripening in women that undergo induction of labour by means of a vaginal prostaglandin slow-release delivery system (Propess®).  Prospective observational study on 204 women that required induction of labour between February 2019 and May 2020 at “La Mancha Centro” hospital in Alcázar de San Juan, Spain. The main variable studied was effective cervical ripening (Bishop score > 6). Using multivariate analysis and binary logistic regression, we created three initial predictive models (model A: Bishop Score + Ultrasound cervical length + clinical variables (estimated fetal weight, premature rupture of membranes and body mass index)); model B: Ultrasound cervical lenght + clinical variables; and model C: Bishop score + clinical variables) to predict effective cervical ripening. All three predictive models obtained (A, B and C) presented good predictive capabilities, with an area under the ROC curve ≥ 0.76. Predictive model C, composed of the variables: gestational age (OR 1.55, 95% CI 1.18–2.03, *p* = 0.002), premature rupture of membranes (OR 3.21 95% CI 1.34–7.70, *p* = 0.09) body mass index (OR 0.93, 95% CI 0.87–0.98, *p* = 0.012), estimated fetal weight (OR 0.99, 95% CI 0.99–1.00, *p* = 0.068) and Bishop score (OR 1.49 95% CI 1.18–1.81, *p* = 0.001), is presented as the model of choice with an area under the ROC curve of 0.76 (95% CI 0.70–0.83, *p* < 0.001). A predictive model composed of the variables: gestational age, premature rupture of membranes, body mass index, estimated fetal weight and Bishop score upon admission presents good capabilities in predicting successful cervical ripening following administration of prostaglandins. This tool could be useful in making clinical decisions with regard to induction of labour.

## Introduction

Cervical ripening is required in patients who present unfavourable cervical conditions prior to undergoing induction of labour (IoL), with the aim of increasing the probability of vaginal birth and reducing induction time^1^. This process can be done using pharmacological methods, such as the use of prostaglandins, or mechanical methods, such as insertion of balloon catheters^2^.

So far, no cervical ripening method has been demonstrated to be clearly superior to another in terms of cervical ripening, labour induction or reducing the risk of caesarean^1–3^. In the meta-analysis published by Chen W et al.^2^ in 2016, the use of vaginal misoprostol was demonstrated to be the most effective method for achieving vaginal birth in 24 h, in comparison with the use of vaginal dinoprostone or balloon catheters, and was associated with a lower rate of caesareans in IoL. However, this method presented a higher rate of uterine hyperstimulation with changes in fetal heart rate in comparison to other methods.

The use of vaginal dinoprostone (PGE2) is one of the most commonly used methods to achieve cervical ripening^4^. Many studies have been published in relation to its use, but few have aimed to determine which factors predict successful cervical ripening when dinoprostone is administered by means of the vaginal delivery system Propess® (Ferring Pharmaceutical, Saint-Prex, Switzerland)^5–7^. Additionally, there is high heterogeneity in these studies’ final results, as no specific distinction is made between the cervical ripening process and induction of labour, and different definitions of successful induction of labour are used^8,9^, making it difficult to compare results and draw definitive conclusions.

Knowing the factors associated with the cervical ripening process will allow us to improve success rates in this procedure and select the right pre-induction method for each patient. The objective of this study is therefore to create a model to predict which patients that undergo induction of labour will present successful cervical ripening, defined as a Bishop score (BS) > 6, through use of the vaginal prostaglandin delivery system (Propess®), independently of the final birth outcome.

## Methods

### Design and selection of subjects

We conducted a prospective observational study. A total of 223 women took part in the study between February 2019 and May 2020 at “La Mancha Centro” hospital in Alcázar de San Juan, Spain. Before collecting the data, we obtained written informed consent from the patients and approval from the hospital's clinical research ethics committee (CEIC), with protocol number 102-C. This study follows the principles of the Hensilki declaration.

The study included all women with single-gestation pregnancy that required IoL with cervical ripening through use of 10 mg vaginal prostaglandin (Propess®). Single-gestation pregnancies between weeks 34 and 41 with unfavourable cervix were included in this study. Multiple births, non-cephalic presentation, fetal malformations and inductions due to antepartum fetal death were excluded for ethical reasons. Cases in which the medication was removed due to changes in fetal heart rate during the cardiotocography (CTG) or in which there was secondary uterine hyperstimulation were also excluded from the study.

### Procedure

Induction of labour in patients with an unfavourable cervix (Bishop ≤ 6) is done following the medical indications described by the Spanish Society of Obstetrics and Gynaecology (SEGO)^10^, which involves placing the slow-release delivery system (Propess®) in the posterior vaginal fornix. The device contains 10 mg prostaglandin, which is released at a rate of 0.3 mg/h in 24 h. Once the device has been placed, CTG is performed on the patient over a period of 2 h. If, after insertion, fetal heart rate falls into categories II or III (according to the system proposed by the National Institute of Child Health and Human Development—NICHD^11^) or a uterine tachysystole is observed (defined as more than five contractions in 10 min), the device is removed immediately. If no changes occur, a CTG is performed at 12 h and at 24 h and the device is removed either when the patient achieves favourable cervical ripening (Bishop > 6) and dilation of 3–4 cm with regular uterine contractions; or after 24 h, regardless of Bishop score. After the cervical ripening process, in cases in which it is required, induction of labour continues with intravenous administration of oxytocin in order to regulate uterine dynamics and move forward in the labour process. Artificial rupture of membranes is performed in women with intact membranes and who, after 24 h of cervical ripening, have not started the active phase of labor, if technically possible.

### Information sources and study variables

To collect the information, a specific record was created including variables from the digitised hospital medical histories, additional information obtained from personal interviews, and clinical assessments in the form of the Bishop score and cervical length measured by ultrasound. The independent variables were sociodemographic, obstetric and neonatal in nature (Table 1). The main variable result was effective cervical ripening (BS > 6) obtained following application of vaginal dinoprostone, regardless of the final outcome of the birth (spontaneous, instrumental or caesarean). The Bishop score prior to IoL (BS) and the cervical length (CL) measured by ultrasound were collected by the gynaecologist in charge of the delivery room on the day of the IoL. To measure CL, the guidelines proposed by the International Society of Ultrasound in Obstetrics and Gynecology (ISUOG)^12^ and the Fetal Medicine Foundation (FMF) were followed^13^.

**Table 1:** Sociodemographic and obstetric variables studied.

Variable	N (%)	Mean (SD)
**Maternal age**		32.8 (5.03)
≤ 35 years	140 (68.6)	
> 35	64 (31.4)	
**Weeks** **of gestation**		
< 37	6 (3)	
37–41	147 (72.6)	
≥ 41	50 (24.5)	
**Nationality**		
Spanish	171 (83.8)	
Other	33 (16.2)	
**Body mass index (BMI)**		24.97 (4.67)
**Number of previous pregnancies**		
1	96 (47.1)	
2	56 (27.5)	
≥ 3	52 (25.6)	
**Number of vaginal births**		
None	131 (64.2)	
1	53 (26)	
2	15 (7.4)	
≥ 3	5 (2.5)	
**Previous caesarean**		
Yes	23 (11.3)	
No	180 (88.2)	
Missing values	1	
**Reason for previous caesarean**		
Failure to progress (FTP)	5 (2.5)	
Failed Induction (FI)	3 (1.5)	
Cephalopelvic disproportion (CPD)	4 (2)	
Non-reassuring fetal heart rate (NRFHR)	6 (2.9)	
Podalic presentation	2 (1)	
Maternal illness	2 (1)	
**Fetal sex**		
Male	108 (52.9)	
Female	96 (47.1)	
**Diabetes**		
No	186 (91.2)	
Pregestational	3 (1.5)	
Gestational insulin dependent	13 (6.4)	
Gestational non-insulin dependent	2 (1)	
**Intrauterine growth restriction (IUGR)**		
No	193 (94.6)	
Yes	11 (5.4)	
**Hypothyroidism**		
No	174 (85.3)	
Pregestational	16 (7.8)	
Gestational	14 (6.9)	
**Hypertensive states in pregnancy**		
No	188 (92.2)	
Chronic HTN	4 (2)	
Gestational HTN	8 (3.9)	
Pre-eclampsia	4 (2)	
**Prepartum estimated fetal weight (EFW)**		3179.87 (621.76)
**Prepartum amniotic fluid index (AFI)**		
< 5	24 (11.8)	
5—25	165 (81.3)	
> 25	14 (6.9)	
**Cervical length prior to induction (CL)**		24.48 (9.14)
**Funnelling**		
Yes	21 (10.3)	
No	183 (89.7)	
**Bishop score upon admission**		2.78 (1.37)
0–1	46 (8.8)	
2–4	140 (68.6)	
5–≤ 6	28 (8.9)	
**Reason for induction**		
Post-term pregnancy	67 (32.8)	
Premature rupture of membranes (PROM)	39 (19.1)	
Gestational diabetes	15 (7.4)	
Maternal illness	11 (5.4)	
Hypertensive states in pregnancy	20 (9.8)	
Hydramnios	14 (6.9)	
Oligohydramnios	12 (5.9)	
Intrauterine growth restriction (IUGR)	14 (6.9)	
Non-reassuring status	1 (0.5)	
Macrosomia	1 (0.5)	
SGA	10 (4.9)	
**Bishop score in dilation**		6.28 (1.89)
**Duration of dilation**		288.80 (231.34)
**Duration of second stage**		83.28 (81.03)
**Type of birth**		
Spontaneous	113 (55.4)	
Vacuum	17 (8.3)	
Spatula	11 85.4)	
Forceps	6 (2.9)	
Caesarean	57 (27.9)	
**Indication for instrumental delivery**		
Shorten second stage	21 (61.76)	
Non-reassuring fetal heart rate (NRFHR)	9 (26.47)	
Maternal illness	1 (2.94)	
Inadequate progress	3 (8.82)	
**Indication for caesarean**		
Failure to progress (FTP)	20 (12)	
Failed induction (FI)	9 (5.4)	
Cephalopelvic disproportion (CPD)	7 (4.2)	
Non-reassuring fetal heart rate (NRFHR)	20 (12)	
**Weight of newborn**		3201.68 (504.7)
**APGAR 1** **minute**		
> 7	189 (92.6)	
< 7	15(7.4)	
**APGAR 5 minutes**		
> 7	202 (99)	
< 7	2 (1)	
**Level of resuscitation**		
No resuscitation	161 (68.9)	
Type I	38 (18.6)	
Type II	1 (0.5)	
Type III	4 (2)	
**Arterial PH**		7.27 (0.06)
**Venous pH**		7.32 (0.05)

### Statistical analysis

Firstly, descriptive analysis was conducted with absolute and relative frequencies for categorical variables and mean with standard deviation (SD) for the quantitative variables.

We then conducted bivariate analysis with all potential variables associated with cervical ripening (Table 2) and we pre-selected those with a *P* value < 0.25 (Lemeshow test^14^), using either the Pearson chi-squared or the Student-Fisher t-test, depending on whether the independent variable was qualitative or quantitative. The next step was to perform multivariate analysis through binary logistic regression using the backward stepwise method in SPSS (Stadistical Package for the Social Sciences) to create three predictive models (Table 3), in which odds ratios (OR) were obtained with respective confidence intervals of 95% (CI).

**Table 2:** Bivariate analysis of the obstetric characteristics and effective cervical ripening.

Variable	Cervical ripening			
	Bishop score ≤ 6	Score bishop > 6	OR CI	*P* value
**Maternal Age**	33.5 (5.10)	32.6 (5.24)	0.97 (0.92–1.02)	0.239
**Weeks of gestation**	39.3 (1.47)	39.7 (1.32)	**1.24 (1.01–1.52)**	**0.037**
**Body mass index (BMI)**	27.0 (5.39)	24.7 (5.14)	**0.91 (0.86- 0.96)**	**0.001**
**Parity**				0.378
Primiparous	71 (54.2)	60 (45.8)	1	
Secundiparous	28 (52.8)	25 (47.2)	1.06 (0.56–2.00)	0.866
Terciparous or more	14 (70.0)	6 (30.0)	0.51 (0.18–1.40)	0.190
**Previous caesarean**				0.330
No	98 (54.4)	82 (45.6)	1	
Yes	15 (65.2)	8 (34.8)	0.64 (0.26–1.58)	
**Fertility treatment**				
None	102 (54.5)	85 (45.5)	1	
Insemination	1 (100.0)	0 (0.0)	NC	1.000
In vitro fertilisation	9 (64.3)	5 (35.7)	0.68 (0.22–2.07)	0.482
**Fetal sex**				**0.028**
Female	61 (63.5)	35 (36.5)	1	
Male	52 (48.1)	56 (51.9)	**1.88 (1.07–3.29)**	
**Diabetes**				0.055
No	99 (53.2)	87 (46.8)	1	**0.045**
Yes	14 (77.8)	4 (22.2)	0.33 (0.10–1.03)	
**Intrauterine growth restriction (IUGR)**				
Yes	7 (63.6)	4 (36.4)	1	
No	106 (54.9)	87 (45.1)	0.70 (0.20–2.46)	
**Hypothyroidism**				0.879
No	96 (55.2)	78 (44.8)	1	
Yes	17 (56.7)	13 (43.3)	0.94 (0.43–2.06)	
**Hypertensive states in pregnancy**				0.111
No	101 (53.7)	87 (46.3)	1	
Yes	12 (75.0)	4 (25.0)	0.39 (0.12–1.24)	
**Prepartum estimated fetal weight (EFW)**	3245.2 (57.73)	3187.8 (45.38)	1.00 (0.99–1.00)	0.470
**Cervical length (CL) before induction**	28.79 (9.57)	23.59 (9.02)	0.93 (0.90–0.96)	** < 0.001**
**Funnelling**				0.865
No	101 (55.2)	82 (44.8)	1	
Yes	12 (57.1)	9 (42.9)	1.08 (0.44–2.70	
**Prepartum amniotic fluid index (AFI)**				
< 5	13 (54.2)	11 (45.8)	1	
≥ 5—25	90 (54.5)	75 (45.5)	1.02 (.43–2.40)	0.972
> 25	10 (71.4)	4 (28.6)	0.48 (0.15–1.59)	0.230
**Bishop score upon admission**	2.16 (1.41)	3.04 (1.35)	1.58 (1.28—1.96)	** < 0.001**
**Premature rupture of membranes (PROM)**				**0.001**
No	101 (61.2)	64 (38.8)	1	
Yes	12 (30.8)	27 (69.2)	**3.55 (1.68—7.50)**	
**Hours between rupture of membranes and induction**	7.6 (6.00)	13.3 (18.16)	1.05 (0.95–1.16)	0.164
**Time with Propess®**	14.5 (7.27)	9.3 (5.31)	**0.88 (0.84- 0.93)**	** < 0.001**

Significant values are in [bold].

**Table 3:** Predictive models for cervical ripening. Multivariable analysis.

Variables	Coef B	aOR IC95%	*P* value	ROC− AUC
**Model A**				
Gestational age	0.463	1.58 (1.20–2.09)	0.001	0.77 (0.71–0.84)
PROM	1.080	2.95 (1.21–7.15)	0.017	
BMI	− 0.066	0.93 (0.88–0.99)	0.035	
Estimated fetal weight	− 0.001	0.99 (0.99–1.00)	0.044	
Bishop score upon admission	0.296	1.34 (1.04–1.73)	0.023	
Cervical length (TVS)	− 0.042	0.95 (0.92–0.99)	0.036	

**Model B**				
Gestational age	0.475	1.61 (1.22–2.11)	0.001	0.76 (0.69–0.82)
PROM	1.269	3.56 (1.50–8.41)	0.004	
BMI	− 0.058	0.94 (0.88–1.00)	0.060	
Estimated fetal weight	− 0.001	0.99 (0.99–1.00)	0.035	
Cervical length (TVS)	− 0.060	0.94 (0.91–0.97)	0.001	

**Model C**				
Gestational age	0.437	1.55 (1.18–2.03)	0.002	0.76 (0.70–0.83)
PROM	1.168	3.21 (1.34–7.70)	0.009	
BMI	− 0.078	0.93 (0.87–0.98)	0.012	
Estimated fetal weight	− 0.001	0.99 (0.99–1.00)	0.068	
Bishop score upon admission	0.404	1.49 (1.18–1.81)	0.001	

Model (A) was composed of pre-selected variables plus BS and CL, model (B) was composed of pre-selected variables plus CL, and model (C) was made up of pre-selected variables plus BS.

The reason that we initially created three predictive models was to be able to compare their predictive capability and to identify which variable is a better predictor of response to cervical ripening with dinoprostone: Bishop score, cervical length, or a combination of both.

The predictive capability was determined using the area under the ROC curve, with respective confidence intervals of 95%. The final model was selected based on three criteria: clinical plausibility, predictive capability, and principle of parsimony (least number of variables). All calculations were done using the program SPSS v24.0.

### Ethical approval

Ethical approval for this study was granted in February 2019 by the hospital’s clinical research ethics committee (CEIC) in Ciudad Real (Spain), with protocol number 102-C.

### Informed consent

Written informed consent was obtained from all women included in the study before collecting the data.

## Results

**Figure 1:**
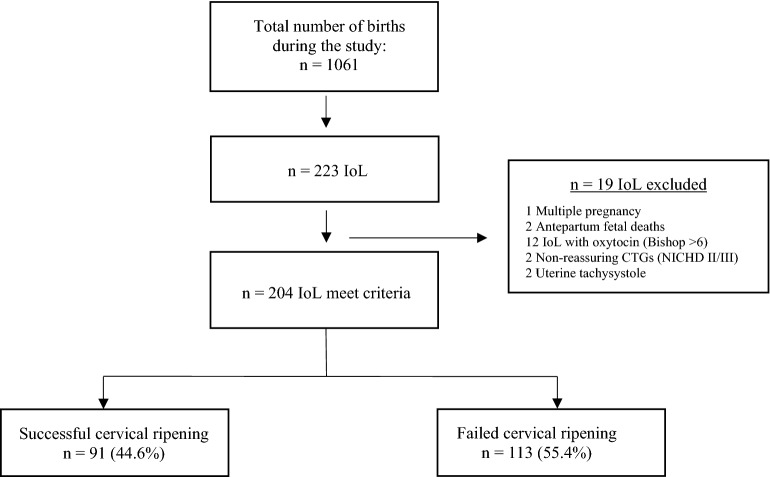
Flow chart of patient selection process.

The total number of patients attended to during the period of study was 1061. Of the 223 inductions, 204 (91.47%) met the inclusion criteria, while 19 (8.52%) women were excluded from the study. Following insertion of the PROPESS, 91 (44.6%) patients achieved successful cervical ripening, while 113 (55.4%) presented a Bishop score < 6. The patient selection process is shown in Fig. 1.

Table 1 contains all of the obstetric and socioeconomic variables analysed. The main reason for induction was post-term pregnancy (67, 32.8%) followed by premature rupture of membranes (PROM) (39, 19.1%) and diabetes (15, 7.4%). The mean Bishop score (BS) was 2.78 (SD = 1.37) and the mean cervical length (CL) measured by ultrasound prior to IoL was 24.48 mm (SD = 9.14).

Next, the relationship between effective cervical ripening and the independent variables was studied by means of bivariate analysis, observing statistical relationships with gestational age (*p* = 0.037), body mass index (BMI) (*p* = 0.001), fetal sex (*p* = 0.028), CL measured by ultrasound prior to IoL (*p* < 0.001), BS upon admission (*p* = 0.001), PROM (*p* = 0.001) and time with Propess® (*p* < 0.001). Table 2 shows the bivariate analysis.

Finally, the multivariate analysis was conducted, obtaining three initial predictive models (Table 3) composed of the following variables: gestational age, premature rupture of membranes (PROM), body mass index (BMI), Estimated fetal weight (EFW), Bishop score (BS) upon admission and cervical lenght (CL) measured by ultrasound.

**Figure 2:**
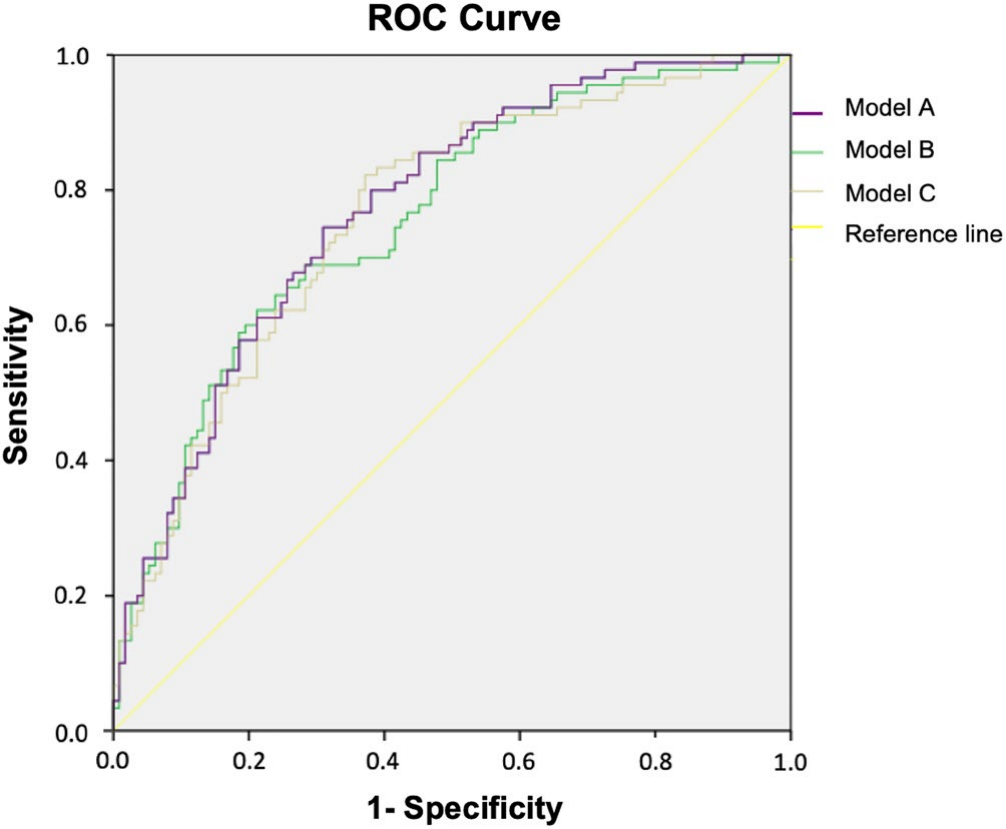
ROC curves for predictive models A, B and C.

Model A presented an area under the ROC curve of 0.77 (95% CI 0.71–0.84, *p* < 0.001). Model B presented a ROC-AUC of 0.76 (95% CI 0.69–0.82, *p* < 0.001). Model C also presented a ROC-AUC of 0.76 (95% CI 0.70–0.83, *p* < 0.001). All three models presented good predictive capabilities, with an area under the ROC curve ≥ 0.76. Figure 2 shows the ROC curves for each model and compares them.

Predictive model C was chosen based on the criteria of clinical plausibility, predictive capability, and parsimony. It was composed of the variables: gestational age (odds ratio [OR]1.55, 95% confidence interval [CI] 1.18–2.03, *p* = 0.002), premature rupture of membranes (PROM) (OR 3.21 95% CI 1.34–7.70, *p* = 0.09) body mass index (BMI) (OR 0.93, 95% CI 0.87–0.98, *p* = 0.012), estimated fetal weight (EFW) (OR 0.99, 95% CI 0.99–1.00, *p* = 0.068) and BS (OR 1.49 95% CI 1.18–1.81, *p* = 0.001).

## Discussion

The variables associated with successful cervical ripening (Bishop score > 6) were: gestational age, PROM and Bishop score upon admission. These results coincide with those reported in the literature in relation to the labour induction process overall^15^. Obesity and estimated fetal weight (EFW), factors widely known to be predictors of failure in IoL^8,16^, were also risk factors for failure in cervical ripening. In contrast, estimated fetal weight (EFW) is not reported to be a predictor of cervical ripening failure in studies conducted by Daykan^6^ and Hiersch^5^.

We did not find any association between successful cervical ripening and maternal age, parity, reason for induction, number of hours between rupture of membranes and placement of vaginal dinoprostone, or neonatal factors.

We only identified two studies in the literature that present models that predict success in the cervical ripening process with vaginal dinoprostone slow-release devices. Hiersch et al.^5^ presented an initial predictive model composed of parity, cervical dilation at admission and gestational age, with a ROC-AUC of 0.79 (95% CI 0.74–0.84) and a second, more complex, model composed of maternal age, BMI, parity, cervical dilation, effacement, indication for induction, gestational age and neonatal weight, with a ROC-AUC of 0.80 (95% CI 0.75–0.85). Melamed et al.^7^ identified maternal age > 30 years, nulliparity, BMI ≥ 25, cervical dilation < 1 cm, effacement ≤ 50% and gestational age > 37 weeks as predictors of failure of cervical ripening, and created a logistic regression model that can predict ≈50% of all cases of failed ripening (R^2^ = O.47).

However, we agree with the conclusions of Melamed et al.^7^ and must be cautious when interpreting results reported in the literature. Most studies analyse successful induction of labour, defined as vaginal birth within 24 h^8,9,17^, as the final result, without distinguishing induction from the prior cervical ripening process, so it is not possible to correctly evaluate cervical response to the action of vaginal dinoprostone without this result being affected by additional intrapartum factors.

With regard to the cervical ripening process, we must clarify that there is no universally accepted threshold score to define a favourable or unfavourable cervix that tells us how to begin an induction. High Bishop scores have traditionally been associated with higher vaginal birth success rates^18,19^. However, there are studies that question the reliability of Bishop scores in predicting the final birth outcome^20^. In our study, we considered the cervical ripening process to be successful after obtaining a BS > 6 with administration of vaginal dinoprostone, basing ourselves on the results obtained in the majority of randomised studies and in clinical guidelines for induction of labour^21,22^.

As for the selection of the most adequate predictive model, many studies have compared the predictive capability of cervical length (CL) measured by ultrasound versus Bishop score on induction of labour outcomes, with conflicting results^23–26^. A systematic review conducted by Cochrane in 2015^27^ did not find significant differences between both methods (CL vs BS) in terms of rates of vaginal births, caesareans and admission into NICU, and concluded that there is not enough evidence to recommend the use of CL over the standard digital examination in assessing cervical ripening.

As well as cervical length (CL) measured by ultrasound, fetal fibronectin has also been studied in assessing cervical ripening, but it has not been found to be superior to the Bishop score^15^. Considering the evidence from the published data and the ease of reproducing it, model C, in which the only added variable was the Bishop score, was our chosen model.

The main limitations of this study are related to the sample size, which is relatively small in comparison to other studies^5,8^. Also, our predictive model is created based on the sociodemographic and obstetric characteristics of a Caucasian/Hispanic population, so it must be validated externally in another type of population before being used.

Additionally, this study only analyses the obstetric and sociodemographic characteristics that have traditionally been associated with success in the labour induction process^15,28^. However, these data must be interpreted with caution, as there is still a certain lack of understanding of the physiological phenomena involved in the onset of labour and cervical ripening, and there is wide biological variation among mothers in the normal labour process^29^.

As far as strong points, we can mention its prospective observational design, making the collection of variables more exhaustive and complete. What's more, all patients included in the study are treated based on a homogeneous labour indication protocol with clear indications on the end of the same, reducing possible biases related to its use.

## Conclusion

In conclusion, successful cervical ripening through the administration of the vaginal prostaglandin slow-release delivery system (Propess®) can be predicted from specific variables: gestational age, BMI, PROM, EFW and BS upon admission, through the use of this predictive model (C). Including this predictive model in hospital labor induction protocols could help in decision-making regarding the indication of this procedure by using the variables that best predict the success of cervical ripening with this induction method.

## Data Availability

The datasets used and/or analysed during the current study available from the corresponding author on reasonable request.

## References

[1] Levine LD (2020). Cervical ripening: Why we do what we do. Semin. Perinatol..

[2] Chen W, Xue J, Peprah MK, Wen SW, Walker M, Gao Y, Tang Y (2016). A systematic review and network meta-analysis comparing the use of Foley catheters, misoprostol, and dinoprostone for cervical ripening in the induction of labour. BJOG.

[3] Zhu L, Zhang C, Cao F, Liu Q, Gu X, Xu J, Li J (2018). Intracervical Foley catheter balloon versus dinoprostone insert for induction cervical ripening: A systematic review and meta-analysis of randomized controlled trials. Medicine (Baltimore).

[4] Shirley M (2018). Dinoprostone vaginal insert: A review in cervical ripening. Drugs.

[5] Hiersch L, Borovich A, Gabbay-Benziv R, Maimon-Cohen M, Aviram A, Yogev Y, Ashwal E (2017). Can we predict successful cervical ripening with prostaglandin E2 vaginal inserts?. Arch. Gynecol. Obstet..

[6] Daykan Y, Biron-Shental T, Navve D, Miller N, Bustan M, Sukenik-Halevy R (2018). Prediction of the efficacy of dinoprostone slow release vaginal insert (Propess) for cervical ripening: A prospective cohort study. J. Obstet. Gynaecol. Res..

[7] Melamed N, Ben-Haroush A, Kremer S, Hod M, Yogev Y (2010). Failure of cervical ripening with prostaglandin-E2 can it be predicted?. J. Matern. Neonatal Med..

[8] Pevzner L, Rayburn WF, Rumney P, Wing DA (2009). Factors predicting successful labor induction with dinoprostone and misoprostol vaginal inserts. Obstet. Gynecol..

[9] Rouzi AA, Alsibiani S, Mansouri N, Alsinani N, Darhouse K (2014). Randomized clinical trial between hourly titrated oral misoprostol and vaginal dinoprostone for induction of labor. Am. J. Obstet. Gynecol..

[10] Sociedad Española de Ginecología y Obstetricia. Inducción de parto. GAP SEGO. 2013 Jun; 1–23.

[11] Parer JT, Ikeda T, King TL (2009). The 2008 national institute of child health and human development report on fetal heart rate monitoring. Obstet. Gynecol..

[12] Kagan KO, Sonek J (2015). How to measure cervical length. Ultrasound Obstet. Gynecol..

[13] Fetal Medicine Foundation. Cervical assessment. www.fetalmedicine.org. Analizable at https://fetalmedicine.org/education/cervical-assessment

[14] Hosmer, D. W., Lemeshow, S. & Sturdivant, R. X. 2013 *Applied Logistic Regression. 3rd Edn, Hoboken*. (Wiley, Newyork).

[15] Crane JM (2006). Factors predicting labor induction success: a critical analysis. Clin. Obstet. Gynecol..

[16] Ennen CS, Bofill JA, Magann EF, Bass JD, Chauhan SP, Morrison JC (2009). Risk factors for cesarean delivery in preterm, term and post-term patients undergoing induction of labor with an unfavorable cervix. Gynecol. Obstet. Invest..

[17] Hou L, Zhu Y, Ma X, Li J, Zhang W (2012). Clinical parameters for prediction of successful labor induction after application of intravaginal dinoprostone in nulliparous Chinese women. Med. Sci. Monit..

[18] BISHOP EH. PELVIC SCORING FOR ELECTIVE INDUCTION. Obstet Gynecol. **24**, 266–268. (1964). 14199536

[19] Teixeira C, Lunet N, Rodrigues T, Barros H (2012). The Bishop Score as a determinant of labour induction success: A systematic review and meta-analysis. Arch. Gynecol. Obstet..

[20] Kolkman DG (2013). The Bishop score as a predictor of labor induction success: a systematic review. Am. J. Perinatol..

[21] Intrapartum fetal heart rate monitoring: nomenclature, interpretation and general management principles. ACOG practice bulletin No 106. American College of Obstetricians and Gynecologists Obstet Gynecol **114**, 192–202. (2009). 10.1097/AOG.0b013e3181aef106 19546798

[22] Leduc D, Biringer A, Lee L, Dy J (2013). CLINICAL PRACTICE OBSTETRICS COMMITTEE; SPECIAL CONTRIBUTORS. Induction of labour. J Obstet Gynaecol Can..

[23] Verhoeven CJM (2013). Transvaginal sonographic assessment of cervical length and wedging for predicting outcome of labor induction at term: A systematic review and meta-analysis. Ultrasound Obstet. Gynecol..

[24] Gabriel R, Darnaud T, Chalot F, Gonzalez N, Leymarie F, Quereux C (2002). Transvaginal sonography of the uterine cervix prior to labor induction. Ultrasound Obstet. Gynecol..

[25] Hatfield AS, Sanchez-Ramos L, Kaunitz AM (2007). Sonographic cervical assessment to predict the success of labor induction: a systematic review with metaanalysis. Am. J. Obstet. Gynecol..

[26] Ben-Harush Y (2016). The use of sonographic cervical length assessment for the prediction of time from induction to delivery. J. Matern. Neonatal Med..

[27] Ezebialu IU, Eke AC, Eleje GU, Nwachukwu CE. Methods for assessing pre-induction cervical ripening. *Cochrane Database Syst Rev.* **12**(6), CD010762. 10.1002/14651858.CD010762.pub2. (2015). 10.1002/14651858.CD010762.pub2 PMC4473357 26068943

[28] Gibson KS, Waters TP (2015). Measures of success: Prediction of successful labor induction. Semin. Perinatol..

[29] Yount SM, Lassiter N. The pharmacology of prostaglandins for induction of labor. *J Midwifery Womens Health.* **58**(2), 133–144 quiz 238–9. 10.1111/jmwh.12022. (2013). 10.1111/jmwh.12022 23590485

